# Editorial: Functional materials with charge transfer properties and their application in photoelectric devices—volume II

**DOI:** 10.3389/fchem.2024.1405774

**Published:** 2024-04-08

**Authors:** Meng Zheng, Yue Liu, Teresa Gatti, Yongtao Qu

**Affiliations:** ^1^ Qingdao Haiwan Science and Technology Industry Research Institute Co, Ltd (HWSTI), Qingdao Haiwan Chemistry Co, Ltd (QHCC), Qingdao, China; ^2^ College of Material Science Engineer, Liaoning Technical University, Fuxin, China; ^3^ Department of Applied Science and Technology, Politecnico di Torino, Turin, Italy; ^4^ Department of Mathematics, Physics and Electrical Engineering, Northumbria University, Newcastle Upon Tyne, United Kingdom

**Keywords:** charge transfer properties, molecular design strategy, structure-property relationships, electronic applications, photoelectric devices

In the second Research Topic of “Functional Materials with Charge Transfer Properties and Their Application in Photoelectric Devices”, we accepted 7 articles which are all dedicated to explore the charge transfer behavior but feature different editorial type, including original researches, perspectives and mini-reviews. It worth noting that, in these papers, the charge transfer mechanism plays always a key role in how the structure and properties of the different kind of materials are tailored: small molecules, polymers and nano-composite materials are reported for potential uses in supercapacitors, as semiconductors, in biosensors, as catalysts and within wearable devices.

## Supercapacitors

I. Kriegel et al. developed a new hybrid model system that has potential for being very convenient in future energy harvesting and storage applications and capable of undergoing multiple cycles of re-use. Indium tin oxide (ITO) nanocrystals (NCs) with photo-induced electron accumulation properties are used in this work not only as a charge provider, but also for a charge storage role, while typical redox mediators were chosen to enhance the light-induced charge accumulation on the ITO-NCs side and control the reversibility of the system. They explored the possible interaction between ITO NCs and the redox mediators, by monitoring the changes in the optical behavior of ITO/redox hybrid systems upon ultraviolet illumination. They demonstrated that the presence of the redox mediator induced a transfer of the photo-generated electrons from ITO, supported by the increase in charge density of the doped metal oxide NCs, thus resulting in a hybrid system with potential exploitability in photocapacitors or solar redox flow battery systems.

T. Gatti et al. reported on 2D transition metal dichalcogenides (TMDs)/polyaniline nanohybrids with good electrical conductivity and capacitive behavior. In their work, they exploited the liquid-phase exfoliation (LPE) method to produce TMD nanoflakes as scaffolds, onto which induce the *in situ* aniline polymerization and thus achieve porous architectures, with the help of surfactants, to favor the mixing of the two materials, and sodium chloride acting as a templating agent to produce a porous architecture. This mixing strategy between the two components, triggered by different compatibilizers and the templating agent was successful to provide peculiar morphological features in the resulting nano-structures. Those species exhibited good capacitive behavior in neutral pH, achieving maximum specific capacitance of 160 F/g at a current density of 1 A/g, demonstrating the attractiveness of similar nanohybrids for future use in low-cost, easy-to-make supercapacitor devices and flexible electronic products.

## Semiconductors

H. Zhang et al. devoted to design intramolecular charge transfer (ICT) molecules and explore their application in organic field effect transistors (OFET) devices. They designed and synthesized linear and branched polymers with donor-acceptor structure. They discovered that the branched polymer exhibited higher hole transfer efficiency (2.3 × 10^−3^ cm^2^v^−1^s^-1^) than the linear one (1.1 × 10^−3^ cm^2^v^−1^s^-1^) although they featured similar conjugated backbones, indicating that molecular weight is more important than molecular aggregation ability when designing organic polymeric semiconductors for OFET. With similar molecular design strategy, they synthesized a π-expanded diketopyrrolopyrrole small molecule named FDPP with good molecular packing and intercharge transport performance, presenting a large red-shift and strong absorption in thin film state. The corresponding OFET device demonstrated p-type behavior with a hole mobility of up to 9.7 × 10^−3^ cm^2^v^−1^s^-1^, suggesting a great potential for paving the way to novel organic semiconductors.

## Biosensors and catalysts

D. Zhang et al. reported fluorescent probes for intracellular PH and biological species detection based on the intramolecular charge transfer mechanism, these fluorescent probes with donor-acceptor structure could endow blue/red shift when connected interaction being formed with the donor/acceptor groups, which contributed to the charge distribution in an electron system with the push-pull effect. The ICT-based fluorescent probes were reviewed to be advisable biosensors in organisms for the application of intracellular PH detection, gas detection, metal ion detection and anion detection.

M. Wang et al. reported a nano-heterostructured composite based on graphene oxide (GO) and modified Montmorillonite (MMT) and proved that the nano-heterostructure increased the effective interface interaction among TiO_2_, GO, and MMT, which increased the charge transfer ability and prolonged the electron-hole separation time, making it a potential photocatalyst to eradicate environmental pollutants.

## Wearable devices

Focus on wearable devices was presented by Z. Deng et al., that reviewed the development of stretchable and self-repairing materials applied to electronic skin. They stated that a structural design strategy based on the charge transfer mechanism is the most powerful approach to achieve flexible electronic devices with high mobility. Introducing flexible conjugated groups into the conjugated backbone and dynamic reversible bonds into the molecular skeleton chain, make it possible to mitigate the brittleness of high mobility semiconductor materials, to the extent that flexible electronic devices such as electronic skin becomes feasible.

